# Vaccination: Developing and implementing a competency-based-curriculum at the Medical Faculty of LMU Munich

**DOI:** 10.3205/zma001004

**Published:** 2016-02-15

**Authors:** B. Vogel, S. Reuter, M. Taverna, M. R. Fischer, J. Schelling

**Affiliations:** 1 Klinikum der Universität München, Institut für Allgemeinmedizin, München, Deutschland; 2 Klinikum der Universität München, Institut für Didaktik und Ausbildungsforschung in der Medizin, München, Deutschland

**Keywords:** medical education, curriculum, competency-based, vaccines, National Competency-Based Learning Objectives Catalogue Medicine

## Abstract

**Background:** In Germany medical students should gain proficiency and specific skills in the vaccination field. Especially important is the efficient communication of scientific results about vaccinations to the community, in order to give professional counseling with a complete overview about therapeutic options.

**Aim of the project: **The aim of this project is to set up a vaccination-related curriculum in the Medical Faculty at the Ludwig-Maximilians-University in Munich. The structure of the curriculum is based on the National catalogue for competency-based learning objectives in the field of vaccination (Nationaler Kompetenzbasierter Lernzielekatalog Medizin NKLM). Through this curriculum, the students will not only acquire the classical educational skills concerning vaccination in theory and practice, but they will also learn how to become independent in the decision-making process and counseling. Moreover, the students will become aware of consequences of action related to this specific topic.

**Methods:** According to defined guidelines, an analysis was performed on courses, which are currently offered by the university. A separate analysis of the NKLM was carried out. Both analyses identified the active courses related to the topic of vaccination as well as the NKLM learning objectives. The match between the topics taught in current courses and the NKLM learning objectives identified gaps concerning the teaching of specific content. Courses were modified in order to implement the missing NKLM learning objectives.

**Results: **These analyses identified 24 vaccination-related courses, which are currently taught at the University. Meanwhile, 35 learning objectives on vaccination were identified in the NKLM catalogue. Four of which were identified as not yet part of the teaching program. In summary, this interdisciplinary work enabled the development of a new vaccination-related curriculum, including 35 learning objectives, which are now implemented in regular teaching courses by the Medical Faculty.

**Conclusions:** This project successfully describes a method to develop and implement a competency-based teaching program on the topic of vaccination. Importantly, the process presented here can serve as a guide to develop and implement similar teaching programs on other subjects and Universities.

## Zusammenfassung

**Hintergrund: **Jeder Absolvent des Medizinstudiums in Deutschland sollte eine Impfkompetenz mit dem entsprechenden Wissen und Fertigkeiten aufweisen. Dazu gehört u.a. wissenschaftliche Ergebnisse in einer für Laien verständlichen Form kommunizieren zu können, um im Sinne der partizipativen Entscheidungsfindung zu den relevanten Impfthemen beraten zu können.

**Zielsetzung: **Ziel dieses Projektes war die Entwicklung und Implementierung eines strukturierten, kompetenzbasierten Impf-Curriculums an der Medizinischen Fakultät der LMU München. In Anlehnung an den Nationalen Kompetenzbasierten Lernzielkatalog Medizin (NKLM) wurden kompetenzbasierte Impf-Lernziele definiert. Durch das Unterrichten der Impf-Lernziele sollen die Studierenden dazu befähigt werden, Impfungen inklusive der Beratung und Vorbereitung selbständig und situationsadäquat in Kenntnis der Konsequenzen durchzuführen.

**Methodik:** Nach fest definierten Impf-Kriterien wurden eine Istanalyse der bestehenden Lehrveranstaltungen und eine vergleichende Analyse des NKLM durchgeführt. Diese Analyse identifizierte die aktuellen Impf-Lehrveranstaltungen sowie NKLM Impf-Lernziele. Die NKLM Impf-Lernziele wurden daraufhin den bestehenden Lehrveranstaltungen zugeordnet und konsekutiv nicht gelehrte NKLM Impf-Lernziele identifiziert. Bestehende Lehrveranstaltungen wurden modifiziert oder neue Lehrveranstaltungen entwickelt, um diese Lernziele zusätzlich zu implementieren.

**Ergebnisse: **Die Istanalyse identifizierte insgesamt 24 Lehrveranstaltungen, die bereits Impf-Themen lehrten. Der NKLM Impf-Lernzielkatalog beinhaltet insgesamt 35 Lernziele, von denen vier zuvor noch nicht in den untersuchten Veranstaltungen gelehrt wurden. In enger, interdisziplinärer Zusammenarbeit wurde ein longitudinales Impf-Curriculum mit insgesamt 35 Impf-Lernzielen entwickelt und implementiert.

**Schlussfolgerung: **Am Beispiel der Gestaltung und Implementierung eines Impf-Curriculums wurde eine Methodik entwickelt kompetenzbasierte Lernziele in enger interdisziplinärer Zusammenarbeit zu implementieren. Diese Methodik kann auch für größere Themenbereiche oder an anderen Universitäten angewendet werden.

## 1. Introduction

Vaccination belongs to the most important preventive measures against infectious diseases. High vaccination rates also protect individuals who decide not to vaccinate. This is important in order to decrease the infection rate in the whole population, and also to create an immunity, which protects individuals, who cannot or do not want to adhere to medical immunization, a sub-population considered to be in special need of protection against dangerous agents in the society [1].

For this reason in 2013 the Coalition Agreement of the Federal Government of Germany stressed the urgency of increasing the vaccination rate [2], especially in regard to measles infection. The political and health-related long-term goal of the European region of the World Health Organization (WHO) is eventually to eliminate totally the viral infection of measles [3]. The WHO indicates the Polio-virus as a successfully controlled example of a highly viral agent in Europe [4]. In particular, vaccination coverage should be increased for health care workers [5]. There appears to be controversy concerning the importance of vaccination and also a lack of vaccination-related information in the community. Such information should be delivered initially from medical doctors. This issue might be caused by a weakness in medical education concerning vaccination. One of the objectives of the national vaccination plan is to provide better implementation of inoculation strategies in order to promote the more effective use of medical doctors in immunizing their patients. The strategy to achieve efficient synergy between medical doctors and patients is to better improve medical education of students during university and beyond [6]. 

The German Scientific Council suggests a competency-based rearrangement of the study program [7]. Competency is intended as the habitual and well established application of communication, knowledge, professional skills, clinical judgment, emotions, data and reflection in the daily practice for the benefit of individual patients and the community [8]. The sometimes controversial competency of vaccination is particularly suitable to be taught to all medical students, who should develop these specific skills during their study. Among these important skills is the capability of communicating scientific data in an easy-to-understand manner to their patients, through comprehensive, open-ended counseling about vaccination.

The Gesellschaft für Medizinische Ausbildung (GMA) und the Medizinische Fakultätenrat (MFT) developed the national competency-based curriculum in medicine called the Nationaler Kompetenzbasierter Lernzielkatalog Medizin (NKLM) [9]. At the beginning of this project, the NKLM was published in a draft form. Following this provisional form, the Institut für Allgemeinmedizin des Klinikums der Universität München developed and implemented a competency-based curriculum on the topic of vaccination for the Medical Faculty at the LMU München in collaboration with the Institut für Didaktik und Ausbildungsforschung. A team of experts now concentrates on developing the curriculum on vaccination which includes five professors, two doctors, one biologist, one public health specialist and one medical student. 

## 2. Description of the project

The approach to develop the new competency-based curriculum on vaccination is oriented toward the model of the Kernzyklus (modified accordingly to Kern) for the curriculum development in the Medical Faculty [10].

The following steps are included:

1. Needs and problem identification
2. Target group
3. Objective goals
4. Strategy and Method
5. Realization/Implementation

### Needs and problem identification

Disease outbreaks, which can be prevented by vaccination, are often related to a lack of attention to vaccinations leading to a high rate of individuals not inoculated [1]. The role of the medical doctor is to identify and advise relevant vaccinations for the patient, accordingly to the patient’s personal needs. Delivering relevant medical information together with an open ended counseling is of major importance. However, the teaching staff at LMU reported a lack of knowledge about the field of vaccination among medical students. 

#### Target group and Objective Goals

A competency-based curriculum for vaccination should prepare students to cope with constantly changing requirements in the handling of patients and in related counseling. The achievement of this goal is now addressed to students of the Medical Faculty of LMU (Munich). 

The following learning objectives were discussed within an expert-team and described accordingly to Guilbert [11]: “At the end of the internship in General Practice the students will manage independently at least one vaccination including the counseling, preparation and the application of the vaccination.”

#### Strategy and Methods

The vaccination curriculum was developed in 2 steps within 6 months. First of all, an analysis of the current teaching program was carried out. Next learning objectives were identified in the NKLM catalogue and subsequently matched to the topics of ongoing lectures. An interdisciplinary team of experts composed of professors, teaching personal, the academic dean, a health research scientist and a medical student took part in the whole project.

##### Analysis of the current teaching program

The team of experts researched the current learning objectives in the 2013 summer term handbook of modules of the Medical Faculty at LMU. They estimated how often a student completing the 9 Semester course, came across vaccination topics during classes and seminars. “Vaccination topics” – accordingly to the module manual of inoculation practice [12] were defined as follows:

- Production of vaccine
- Effectiveness of vaccine
- Recommendation for the vaccine (Ständige Impfkommission STIKO)
- Execution of vaccine (including patient safety instructions)
- Ideological position
- Evaluation of the vaccine trials
- Legal basis (vaccination damage, sickness in case of vaccine resistance)

##### Identification of NKLM learning objectives and alignment to the objectives in the current teaching program

Subsequently, the team of experts extrapolated the learning objectives on vaccination from the NKLM catalogue. A qualified discussion decided upon inclusion or exclusion of discordant learning objectives. The result was a catalogue of NKLM learning objectives on vaccination. The catalogue included 35 learning objectives on vaccination. In addition a medical doctor specialized in general practice and specifically in the field of vaccination, developed a new curriculum about vaccination. The content of this experience-based curriculum was matched with the topics of the NKLM learning objectives on vaccination showing that the experience-based curriculum is only one part of the newly developed NKLM curriculum. Afterwards the NKLM learning objectives on vaccination were matched to the topics of the current teaching program to address gaps in the current teaching program.

#### Realization/Implementation

Comparing the NKLM catalogue to the current LMU curriculum led to the identification of learning objectives which were not yet part of the present teaching program. To overcome this issue, the missing topics were implemented in the teaching by modifying LMU courses.

## 3. Results

### Actual State

**Figure 1:**
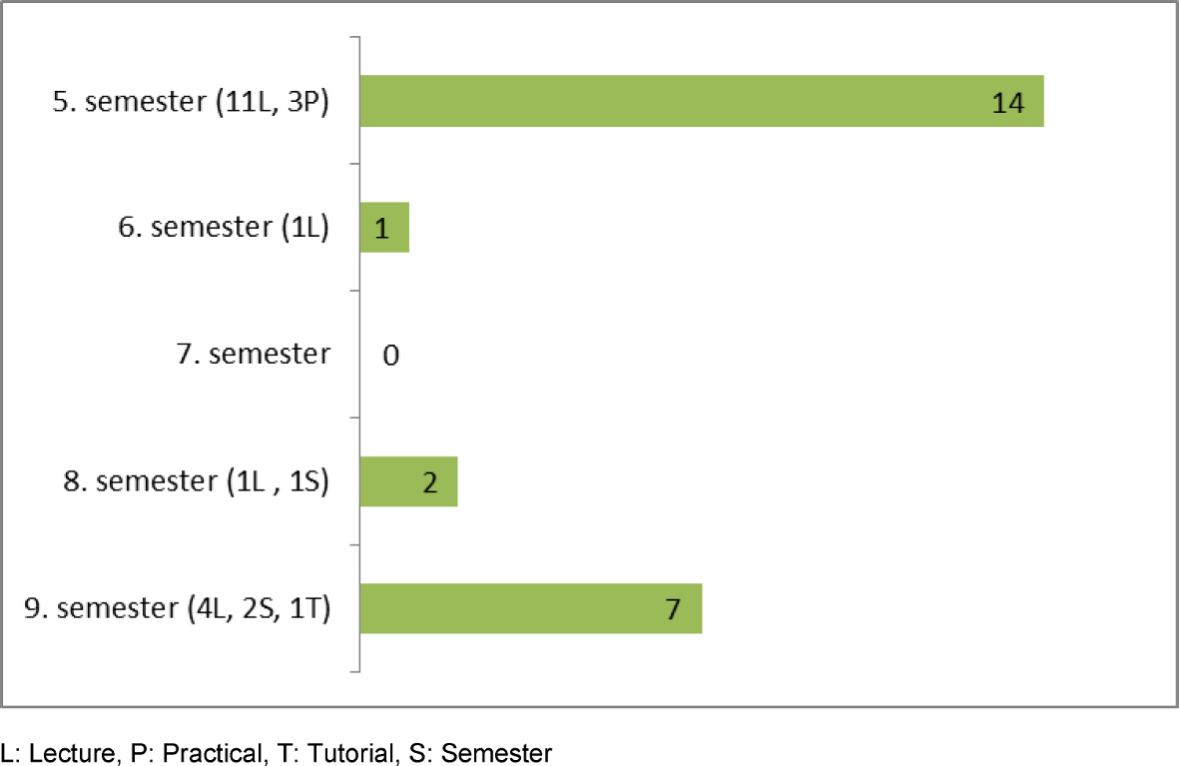
Number of vaccination courses at the Medical Faculty of LMU Munich

The analysis of the teaching modules at the LMU identified 24 lectures about vaccination topics (see Table 1 (Tab. 1)). Moreover, a vaccination is regularly performed during the general practice internship as part of the curriculum. These courses are offered longitudinally during the medical education in the clinical part of the curriculum (see Figure 1 (Fig. 1)).

#### Identification of NKLM- learning objectives as compared to the objectives in the current teaching program.

**Figure 2:**
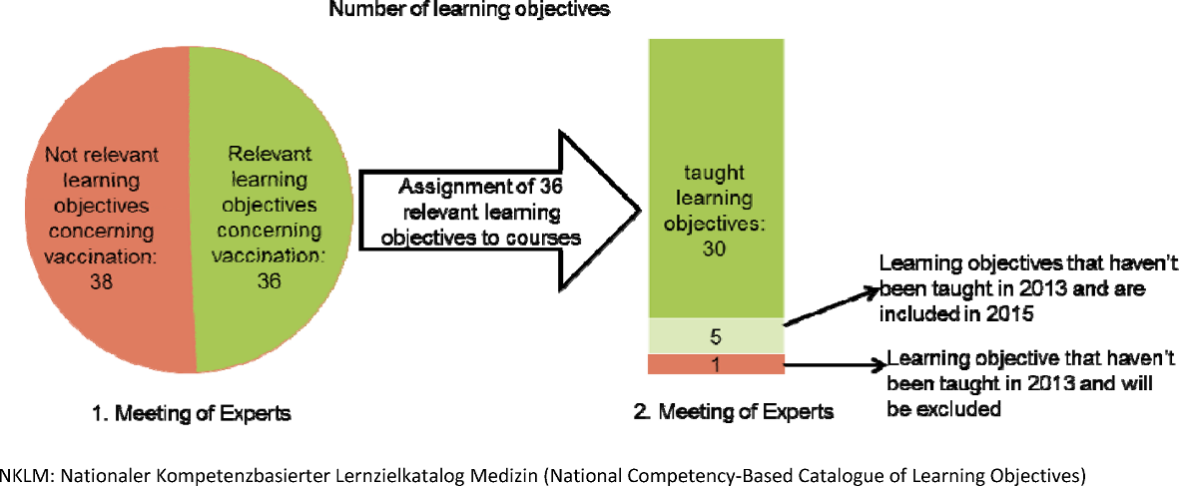
Selection process of NKLM vaccination learning objectives

The first screening of the NKLM identified 74 learning objectives on vaccination. Subsequently, the team of experts agreed unanimously that 35 out of 74 vaccination learning objectives were absolutely relevant for the vaccination lectures. The match between the NKLM learning objectives and the current list of topics taught during lectures revealed that 6 out of 36 objectives were not yet part of the teaching program. In discussions among the members of the teaching commission, one of the 6 objectives was excluded from the list (see Figure 2 (Fig. 2)). A second objective was located within a specific lecture already present in the program (see Table 2 (Tab. 2)). The NKLM catalogue contains 35 learning objectives (see Attachment 1). 

##### Analysis of the NKLM learning objectives on vaccination

**Figure 3:**
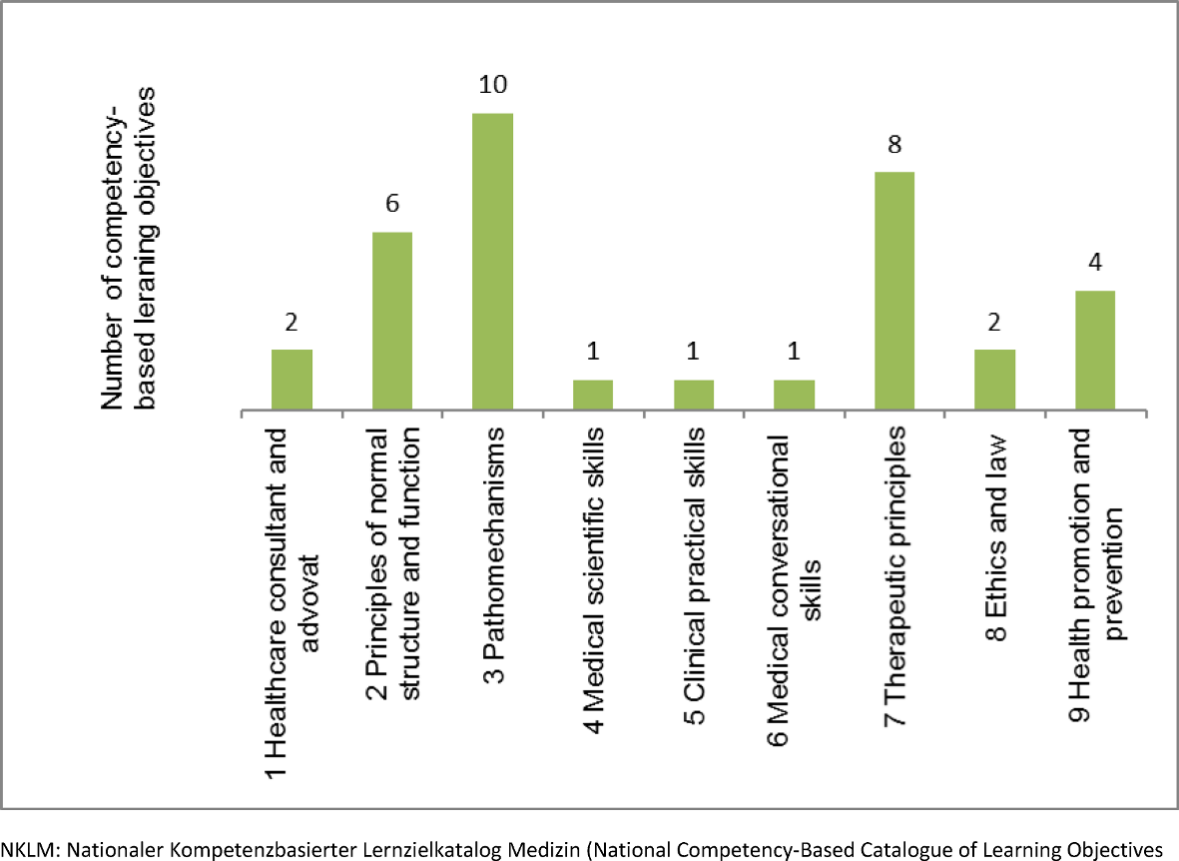
Number of NKLM learning objectives: Roll of competence (1), medical expertise (2-9)

**Figure 4:**
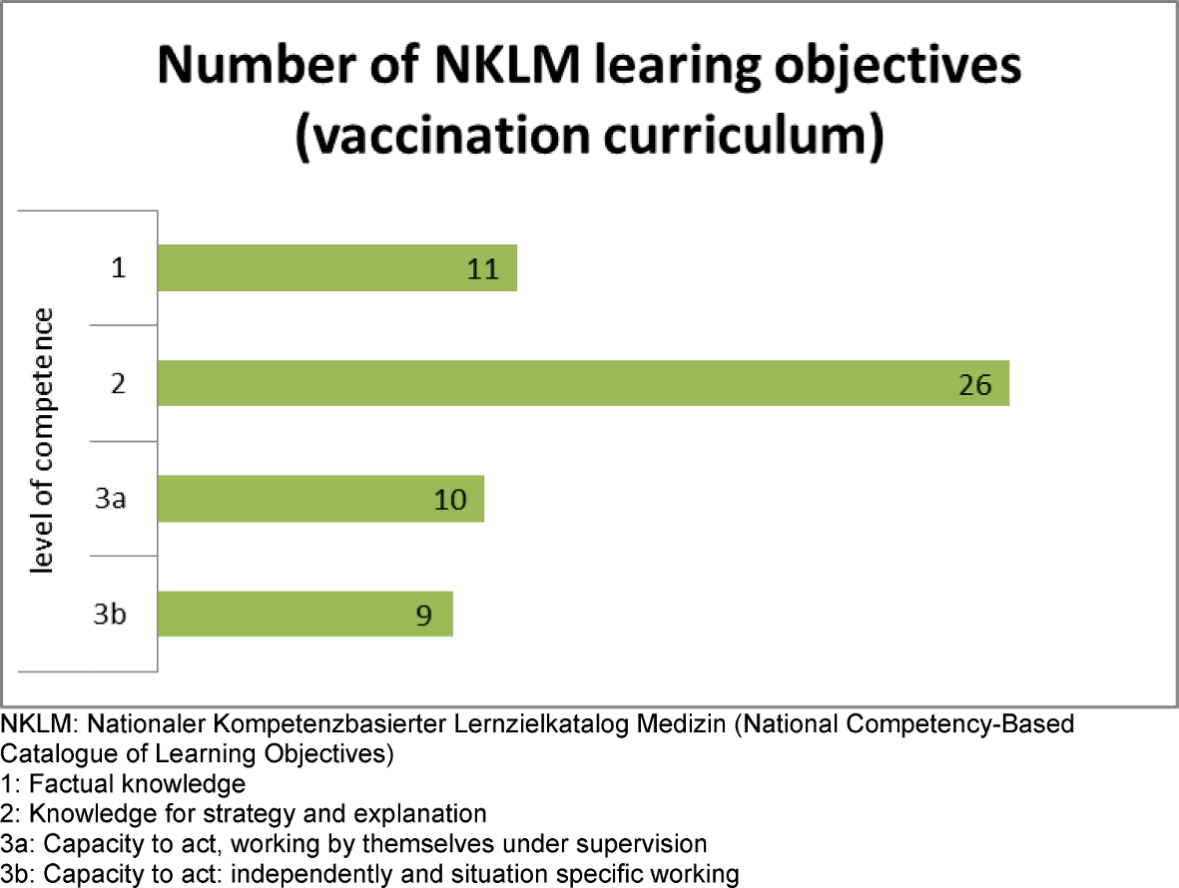
Level of competence and number of NKLM learning objectives of the vaccination curriculum

The implementation of the 35 NKLM learning objectives on vaccination (NKLM vaccination catalogue) was analyzed according to the following topics of the NKLM: roles of competency and medical expertise (see Figure 3 (Fig. 3)). Moreover the NKLM learning objectives on vaccination were classified according to the level of competence. Specifically, the NKLM vaccination curriculum not only desires to expand knowledge competency (Level 1 and 2) but also wants to build the capacity to act properly (Level 3a and 3b) (see Figure 4 (Fig. 4)). So the NKLM vaccination curriculum indicates the following: contents, the point of teaching, and the level of competence.

#### Implementation

31 out of 35 learning objectives from the NKLM catalogue about vaccination were already part of the current teaching program, while 4 of them were found to still be missing. The current teaching courses were modified to include the missing learning objectives. The missing objectives were integrated in different forms within the teaching program: 3 were included in seminars, and 1 was included in a lecture (see Table 2 (Tab. 2)). The teaching format “seminar” reflects the competency-oriented learning-objectives. Moreover, to strengthen the learning process, high value was given to the pre- and post- interactive E-Learning activities in preparation for the attendance-phase. These kinds of activities were integrated into the curriculum. A practical vaccination training, including counseling, preparation, and execution of a vaccination is now part of the practice in family medicine at the end of the students’ medical studies. 

In conclusion, the newly developed NKLM curriculum about vaccination in the Medical Faculty at LMU now contains all 35 learning objectives all integrated into the new teaching program. 

## 4. Discussion

A method to develop a competency-oriented curriculum based on the NKLM was carried out in a number of steps. After performing an analysis of the present teaching program, the learning objectives concerning vaccination were identified. Subsequently, the current learning objectives from the LMU teaching program were matched with the NKLM learning objectives. The next step identified missing learning objectives which were afterwards integrated into the teaching program. Since the educational activities are modified and new learning objectives are now fixed, a reduction of the course content could take place. However, it was important to ensure the teaching of all topics, including several that were outside of the new reassessment of the teaching program. For example, it was possible to substitute the topic “Cough” with the topic “Vaccination”, given that the new topic included basic competences like counseling and a background about the decision process. In this case, although the topic “Cough” was replaced by the topic “Vaccination”, the learning objectives on counseling skills, were still delivered within the courses. 

This analysis of the teaching program revealed to conclusions about lectures concerning the number, type, level of competence, and timing of the lectures. The analysis also elucidated redundancy. These results have been shown in a Curricular Mapping. 

The subject vaccination is now part of the longitudinal program at the LMU, and it covers all the teaching levels. Competences such as scientific skills, which were not yet part of the program, were integrated into the teaching through changes in the curriculum. These methods used for the topic vaccination can be applied as well for other comprehensive topics in medical education. 

The identification of learning objectives in the NKLM for a specific topic served as motivation for the team to carefully screen the NKLM catalogue. The team became aware of the rigidity of the NKLM catalogue. Some believed that the NKLM learning objectives do not promote freedom of inquiry in research and teaching. For this reason, a discussion concerning the relevance of the NKLM learning objectives catalogue arose. It was suggested that the NKLM could serve better as a fixed framework for the preparation of a catalogue with learning objectives. The lecturer can feel free to adopt NKLM learning objectives in detail or individually formulate their own learning objectives including them within the NKLM objectives. Therefore this method can be adjusted to individual parameters in local curricula still insuring fixed standards.

## 5. Conclusions

This paper describes a new approach for the development of competency-based curriculum on the topic vaccination. Our work showed the teaching process can be improved through the determination of competency-oriented learning objectives, the clear assignment of responsibility, and the integration of missing topics. Moreover, this approach can contribute to transparency in the teaching program. 

After the development of the new curriculum about vaccinations, the new catalogue with the updated learning objectives will be evaluated. For example, professors will be asked about the utility of the newly produced catalogue containing the learning objectives, asking them whether it was useful for setting up their list of the topics to teach during the lectures. Moreover, the students will be asked about the NKLM learning objectives. They will be asked whether the new teaching program is helpful toward their preparation for the final exam in general practice. 

The project revealed a dynamic method, for new curriculum development. After positive evaluation results, this method can be applied not only for small topics such as “vaccination,” but also for extended subjects in other universities, in order to develop and implement competency-based learning objectives catalogues. 

## Acknowledgement

The authors acknowledge the constructive contribution during the preparation of the competency-based curriculum for teaching of the topic vaccination provided by Prof. Dr. med. Bogner, Prof. Dr. med. Eberle, Prof. Dr. med. Hübner, Prof. Dr. med. von Kries, PD Dr. rer. nat. Obst and Dr. med. Schrörs.

## Competing interests

The authors declare that they have no competing interests.

## Erratum

The German title was supplemented with the word "Impfen".

## Supplementary Material

Part of NKLM Catalogue

## Figures and Tables

**Table 1:**
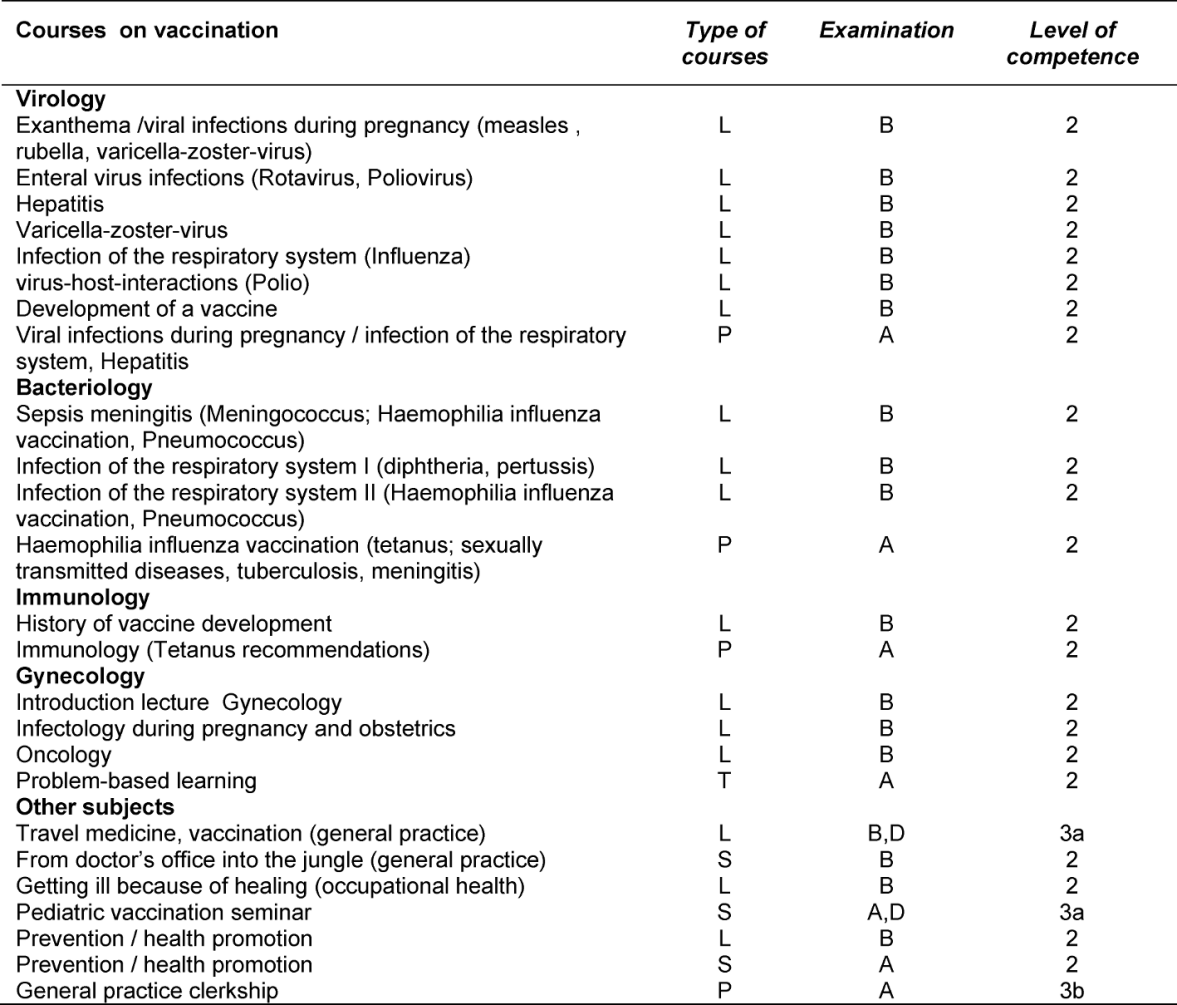
Teaching program on vaccination at the Medical Faculty of LMU Munich summer semester 2013

**Table 2:**
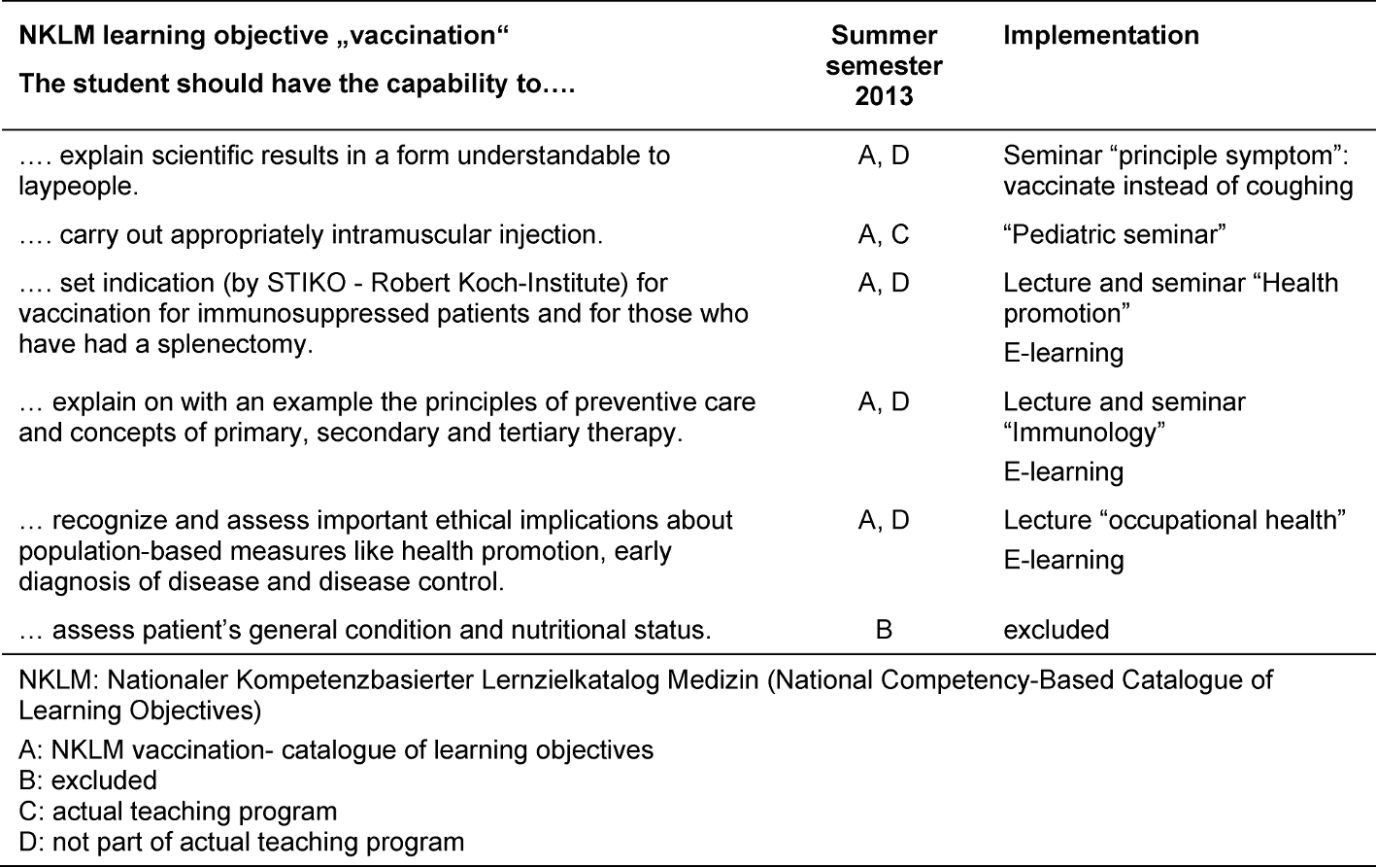
Additional learning objectives
