# Supplementary material for: Vaccination: Developing and implementing a competency-based-curriculum at the Medical Faculty of LMU Munich
Source: GMS J Med Educ. 2016 Feb 15;33(1):Doc5. doi: 10.3205/zma001004 (PMC4766931; doi:10.3205/zma001004)
Supplement: Part of NKLM Catalogue [file JME-33-5-s-001.pdf]

Catalogue of NKLM vaccination learning objectives

NKLM: Nationaler Kompetenzbasierter Lernzielkatalog Medizin (National Competency-Based Catalogue of Learning Objectives)

Competency, learning objective<br>The students should have the capability to…..
… know medical, educational, normative-regulatory and socio-economic capabilities to promote healthy living and health.
… explain medical, educational, normative-regulatory and socio-economic capabilities to promote the state of health of people and populations.
…explain the function und regulation of the cellular and the humoral response.
… describe the molecular and cellular components of humoral and cellular immune system and explain the function.
… explain the antibody’s und T cell receptors’ development and diversity.
… explain the importance of clonal selection and deletion.
… differentiate motive, motivation, intention, volition and activity and examine the relationship among them.
… describe motivational conflicts and the effect on the behaviour.
… describe the basics of Virology.
… explain the viral organ tropism on the basis of surface structure of viruses and affected host cell.
… explain the oncogenes of tumour viruses on the basis of the function of viral proteins and their interaction with host cell’s regulation mechanisms.
… know the viral structure and pathomechanism. Therefore they can conclude the reactions of the immune system after viral infection.
… describe the basics of bacteriology.
… deduce symptoms of bacterial infections on the basis of bacterial Exo- and Endotoxin damaging mechanisms.
… explain the meaning of anthropoids as intermediate hosts for microorganisms (virus, bacteria) or parasites (protozoa, heminths).
… appoint the main mechanism of adaptation of immune reaction against pathogens or body's own proteins and cells and collect risk factors for infections.
… distinguish somatic, psychosocial, socio-cultural and psychological aspects of behaviour and define limits between healthy and ill persons.
… realise effects of immune cells apoptosis as a risk for infection.
… explain scientific results in a understandable form to laypeople.
… carry out appropriately intramuscular injection.
… are familiar with the risks versus the benefits of medical interventions (including screenings, vaccinations and therapies) communicate them in an open and transparent manner.
… explain with an example the principles of preventive care and concepts of primary, secondary and tertiary therapy.
… explain the characteristics of elderly and take into account that the characteristics of elderly influence the therapy.
… explain the characteristics of children and juveniles and take into account that the characteristics of children and juveniles influence the therapy.
…. explain the characteristics of pregnant women and take into account that the characteristics of pregnant women influence the therapy.
… explain the principles of recommended standard vaccinations, (by STIKO - Robert Koch Institute) and carry out the appropriate immunizations.
…. set indication (by STIKO - Robert Koch Institute) for vaccination of immunosuppressed patients and for those who have had a splenectomy.
... apply the principle of therapeutic and prophylactic measures of medications with antiviral and antibacterial properties depending on the organ of interest, the type and severity of the disease, and to prescribe the correct therapy accordingly to the specific infectious clinical case.

… explain the principles and the current significance of individualizing drug therapies .

… practice informed consent.

… recognize and assess important ethical implications of population-based measures like health promotion, early diagnosis of disease und disease control.

… list travel medicine risks of infection and preventive measures.

… list the diseases which are preventable by vaccination with their side effects, use, risks, and legal reasons of the vaccination. They perform detailed Inoculation advice based on current information as well as giving the appropriate vaccinations.

… explain the measures and contents of regular medical checks during pregnancy, for children and juveniles.

… list the current recommendations to manage infectious diseases with children and juveniles and explain the inoculation advices to prevent infectious diseases.
